# Reducing Anticholinergic Burden in Hospitalised Older Adults: Analysis From the INFAR Study

**DOI:** 10.1111/bcpt.70160

**Published:** 2025-12-04

**Authors:** Romana Santos Gama, Luiz Carlos Passos, Welma Wildes Amorim, Renato Morais Souza, Marcio Galvão Oliveira

**Affiliations:** ^1^ Postgraduate Program in Pharmaceutical Services and Policies Federal University of Bahia Salvador Bahia Brazil; ^2^ Department of Internal Medicine, Postgraduate Program in Medicine and Health Federal University of Bahia Salvador Bahia Brazil; ^3^ Department of Health Sciences State University of Southwest Bahia, Vitória da Conquista Campus Vitória da Conquista Bahia Brazil; ^4^ Sarah Kubitschek Rehabilitation Centre Brasília Brazil; ^5^ Multidisciplinary Institute in Health–Anísio Teixeira Campus Federal University of Bahia Vitória da Conquista Bahia Brazil

**Keywords:** anticholinergic burden, clinical pharmacy, deprescribing, medication review, older adults

## Abstract

This study aimed to evaluate the effectiveness of pharmacist‐led medication reviews in reducing the anticholinergic burden of hospitalised patients with cardiovascular diseases (CVDs), to identify which anticholinergic medications were most frequently prescribed or discontinued during hospitalisation and to investigate factors associated with an elevated anticholinergic burden via secondary analysis of the INFAR study. An uncontrolled before‐and‐after design was used, and medication reviews were performed for all patients so that the anticholinergic burden of medications prescribed to older adults during their hospital stay could be examined. The mean age of the 319 patients was 68.9 years (±6.2), and upon hospital admission, 40.4% were classified as having a high anticholinergic burden, decreasing to 22.6% at discharge. Multivariate analysis at admission indicated that polypharmacy (PR = 2.00; 95% CI = 1.47–2.72) and potentially inappropriate medication (PR = 4.47; 95% CI = 2.10–9.53) were independently associated with a higher anticholinergic burden; however, only inappropriate medication was significantly associated with a high burden (PR = 2.39; 95% CI = 1.54–3.71) at discharge. The results indicate that a clinical pharmacist‐led medication review may reduce the anticholinergic burden in older adults with CVD, highlighting the importance of such a review in promoting safer prescribing practices during hospitalisation.

## Plain Language Summary

This study examined whether pharmacist‐led medication reviews can help reduce the use of drugs that have anticholinergic effects, which may be harmful for older adults with heart disease. We assessed 319 hospitalised patients and found that the number with a high anticholinergic burden dropped from 40% at admission to 23% at discharge. Medicines considered inappropriate for older adults were strongly linked to a higher burden. Our findings show that pharmacist involvement during hospital stays can make medication use safer for older patients.

**Trial Registration:** ClinicalTrials.gov: NCT04800900

## 1 Introduction

The increased prevalence of cardiovascular diseases (CVDs) among older adults is associated with a combination of age‐related physiological changes, including oxidative stress, chronic inflammation, cellular apoptosis and progressive vascular and myocardial dysfunction [1]. Additionally, this population frequently presents with multiple comorbidities such as frailty, obesity and diabetes, further increasing the risk of adverse health outcomes. Older adults are also disproportionately affected by polypharmacy and the use of potentially inappropriate medications, including those with anticholinergic properties [1, 2, 3]. These factors contribute to greater vulnerability to medication‐related complications, including anticholinergic burden, which is a critical issue in geriatric pharmacotherapy [3, 4].

Physiological changes that are associated with ageing, such as altered pharmacokinetics and pharmacodynamics, reduced renal function and increased permeability of the blood–brain barrier, enhance the sensitivity of older adults to drug effects and predispose them to drug–drug interactions and other adverse outcomes [2, 5, 6].

Anticholinergic medications are commonly used to treat Parkinson's disease, psychotic disorders, depression, overactive bladders, asthma, allergies and other acute and chronic conditions [4, 6, 7]. These drugs act by inhibiting acetylcholine, a neurotransmitter essential to learning, and can result in central adverse effects, such as agitation, confusion, delirium, hallucinations and cognitive dysfunction, and peripheral effects including urinary retention, constipation, dry mouth, tachycardia and blurred vision [8, 9, 10].

Older adults are particularly vulnerable to the cumulative impact of anticholinergic medications owing to common multimorbidities, age‐related physiological changes and polypharmacy. The concept of the anticholinergic burden refers to the cumulative effect of one or more medications with anticholinergic activity, even if each medication has only mild anticholinergic potential [4, 6, 11]. Over 600 medications have been identified as having anticholinergic effects [11], and this burden is particularly significant in older populations, where 20%–40% of older adults are exposed to polypharmacy [4] and between 33% and 47% are prescribed one or more medications with anticholinergic effects [12]. This cumulative burden has been strongly associated with an increased risk of functional and cognitive decline, falls, hospitalisation, institutionalisation and mortality [3, 4, 6, 11]. Moreover, several studies have demonstrated a link between anticholinergic drug use and an elevated risk of cardiovascular events and mortality [13, 14, 15, 16].

Pharmacist‐led medication reviews consistently reduced anticholinergic burden and the use of inappropriate medications among hospitalised older adults in various countries. Moreover, pharmacists' recommendations were widely accepted by physicians [12], reinforcing the effectiveness and clinical feasibility of these interventions, although studies evaluating safety and long‐term outcomes are still lacking. In addition, there are no data on such interventions in South America.

The present study aimed to evaluate the effectiveness of pharmacist‐led medication reviews in reducing the anticholinergic burden of hospitalised patients with CVDs, to identify which anticholinergic medications were most frequently prescribed or discontinued during hospitalisation and to investigate factors associated with an elevated anticholinergic burden.

## 2 Method

### 2.1 Procedures

#### 2.1.1 Study Design

This study is a secondary analysis of the INFAR study (Effectiveness of Pharmaceutical Interventions in Hospitalised Patients; ClinicalTrials.gov identifier NCT04800900), a before‐and‐after uncontrolled study [17]. The study was based on the review of medications prescribed to patients upon admission and during hospitalisation. Medication reviews were performed by clinical pharmacists; while the INFAR study evaluated broader interventions related to inappropriate prescribing, such as prescribing omissions and the use of potentially inappropriate medications, the specific analysis focused on the anticholinergic burden data of medications prescribed to older adults throughout their hospital stay.

#### 2.1.2 Population

Participants included in the analysis were aged 60 years or older with cognitive capacity, diagnosed with CVDs and hospitalised for more than 24 h during the period March 2021 to January 2022 at Ana Nery Hospital, which is recognised as a reference institution for CVD care in the public health system of Bahia, Brazil. Patients who died in hospital were excluded because the study focused on the prescription process. Participants were recruited from medical wards 24 h after hospitalisation, and data describing prescribed medications were collected at both admission and discharge.

#### 2.1.3 Outcome

Anticholinergic burden at admission and discharge was assessed using the Brazilian Anticholinergic Activity Scale, which assigns scores from 1 to 3 to each drug based on anticholinergic activity. The scale includes 125 drugs that are marketed in Brazil, selected from the ATC classification, 2015 Beers Criteria and other validated scales. Of these, 45 scored 3, 13 scored 2 and 67 scored 1 [6].

The prescriptions of all patients were analysed 24 h after admission and before discharge. Patients were categorised into three groups based on a total score that reflects the cumulative anticholinergic activity of all medications prescribed: no burden (score = 0), low burden (score = 1–2) and high burden (score ≥ 3). A single prescription from each patient was assessed at both time points, guaranteeing an identical sample size for comparison. Data were obtained from interviews, and clinical information and prescriptions from medical records. All data were entered into the KoBoToolbox platform.

#### 2.1.4 Variables of Interest

Several key variables were considered in the INFAR study. Polypharmacy was defined as the prescription of five or more medications, whereas excessive polypharmacy involved 10 or more prescriptions [18]. The Age‐Adjusted Charlson Comorbidity Index (ACCI) was used to assess the burden of comorbidities, with age incorporated into the analysis to predict mortality and survival rates. The result was determined by summing the comorbidity weights and adding an extra score for each decade, starting at the age of 50. A higher ACCI score corresponds to a lower estimated 10‐year survival rate, ranging from 96% to 0% depending on the score [19].

Cognitive impairment was assessed using the Mini‐Mental State Examination (MMSE) [20], a screening tool for evaluating the progression of cognitive disorders, with scores ranging from 0 to 30. Cutoff points were adjusted based on education level, as the threshold for abnormalities varies with academic attainment. Independence in daily activities was evaluated using the adapted Katz Index of Independence in Activities of Daily Living, which examines the ability of an individual to perform daily tasks such as dressing, mobility, feeding and attending to physiological needs [21].

Potentially inappropriate medication was assessed using the MPI Brasil application. This app originated from the Brazilian Consensus on Potentially Inappropriate Medications for Older People [22] and was designed to establish Brazilian‐specific criteria by validating the 2006 and 2012 versions of the STOPP and Beers Criteria. For this study, the second version of the app was employed, which incorporates the 2014 (STOPP) and 2019 (Beers) updates. The MPI Brasil app serves as a comprehensive resource for potentially inappropriate medication evaluation, outlining the rationale for classifying medications as inappropriate, specifying exceptions and suggesting safer therapeutic alternatives, deprescribing strategies and monitoring protocols for unavoidable potentially inappropriate medication use, all accessible via a convenient search function [23].

#### 2.1.5 Study Procedures

Four clinical pharmacists from the hospital staff underwent training focused on the effective management of geriatric patients and the execution of medication reviews. This training incorporated the use of tools designed to improve the assessment of appropriate prescribing, including a checklist featuring the Portuguese‐translated START criteria [24] and the MPI Brasil app.

#### 2.1.6 Medication Review

The primary goal of the medication review was to evaluate and optimise the overall medication regimen of patients. To achieve this, all patient medications were identified, including prescribed drugs, over‐the‐counter medications, herbal supplements and vitamins, and further information was collected through patient interviews, medical records and occasional discussions with family members and caregivers. Each medication was assessed for appropriateness, effectiveness, safety and potential adverse effects while considering the health status and age‐related physiological changes in the patient, along with other relevant factors. Particular attention was paid to medications that could worsen heart conditions or negatively interact with other treatments. Recommendations such as introducing new therapies, discontinuing unnecessary or potentially harmful medications, adjusting dosages to maximise treatment effectiveness and safety, and especially necessary interventions were then made to the attending medical team. The final decision to implement or decline these recommendations rested with the attending physicians, who could choose whether or not to accept the pharmacists' advice. Continuous monitoring was conducted throughout the hospitalisation period, including in instances where patients were transferred to isolation wards or the Intensive Care Unit, and modifications were made to accommodate the changing clinical status, ensuring that all interventions remained safe and effective.

#### 2.1.7 Statistical Analysis

Descriptive analyses of the variables were performed via the creation of two analytical models, with the dependent variables determined based on the anticholinergic burden. Associations between categorical variables were assessed using the chi‐squared test, and the prevalence ratio (PR) was measured to estimate the strength of the association. Differences between the mean values for anticholinergic burden before and after the interventions were assessed using the Wilcoxon nonparametric test, because the Shapiro–Wilk test indicated that the numerical variables were not normally distributed. The R statistical software (R Foundation for Statistical Computing, Vienna, Austria) was used to calculate the PR, whereas all other analyses were performed using the Statistical Package for the Social Sciences version 28.0 (IBM; Armonk, NY, USA). For regression analyses, anticholinergic burden was dichotomised into low/moderate burden (scores = 1–2) and high burden (scores ≥ 3). We fitted Poisson regression models with robust variance to estimate crude and adjusted PRs and their 95% confidence intervals. Variables with a *p*‐value of < 0.20 in univariate analyses were entered into the multivariable models. Patients who had no anticholinergic burden or polypharmacy were excluded from the univariate and multivariate analyses. Consequently, 302 and 258 patients were included in the admission and discharge analyses, respectively. All regression analyses were conducted using complete case analysis.

#### 2.1.8 Ethics Statement

This study was approved by the appropriate institutional review board (approval number: 4.572.301). Written informed consent was obtained from all participants included in this study, and the use of assent forms was not required. The study was conducted in accordance with the Basic and Clinical Pharmacology and Toxicology policy for experimental and clinical studies [25].

## 3 Results

Of the 332 patients evaluated, 13 were excluded because of death, leaving a final sample of 319 individuals. The mean age of the patients was 68.9 years (±6.2), and it comprised 60.2% males, with 85.9% self‐identifying as having black or brown skin. According to the ACCI, 71.5% of patients had a high comorbidity index (ACCI > 2), with 40.1% considered to have an estimated 10‐year survival rate of ≤ 53%. Orthostatic hypotension was the most frequently mentioned self‐reported symptom, affecting 24.1% of patients (Table 1).

**TABLE 1:** Sociodemographic and clinical characteristics of the study population (*n* = 319).

Characteristics	*N* (%)
Sex	
Male	192 (60.2)
Literacy	
Literate	244 (76.5)
Skin colour	
Other (such as black and brown)	274 (85.9)
Smoking	
Smoker and former smoker	214 (67.1)
Alcohol consumption	
Yes	221 (69.3)
Cognitive impairment	
No	274 (85.9)
Poor self‐rated health	
Yes	229 (71.8)
Poor self‐rated memory	
No	226 (70.8)
ADL impairment	
No	285 (89.3)
Delirium	
No	314 (98.4)
Needs assistance for transfer	
No	309 (96.9)
Incontinence	
No	294 (92.2)
Needs assistance for eating	
No	316 (99.1)
Orthostatic hypotension	
No	242 (75.9)
Syncope	
No	271 (85.0)
Constipation	
No	261 (81.8)
History of peptic ulcer	
No	285 (89.3)
Urinary tract symptoms	
No	304 (95.3)
Medication use before admission	
Yes	285 (89.3)
Inappropriate prescribing at admission	
Yes	253 (79.3)
Polypharmacy type	
No polypharmacy	5 (1.6)
Polypharmacy	147 (46.1)
Excessive polypharmacy	167 (52.4)
Charlson Comorbidity Index age‐adjusted	
96% survival in 10 years	20 (6.3)
90% survival in 10 years	71 (22.3)
77% survival in 10 years	100 (31.3)
53% survival in 10 years	59 (18.5)
21% survival in 10 years	34 (10.7)
2% survival in 10 years	17 (5.3)
0% survival in 10 years	18 (5.6)

Abbreviation: ADL: activities of daily living.

Concerning the anticholinergic burden, 4.4% of patients presented with no burden at hospital admission, 55.2% had low burden, and 40.4% were classified as high burden. At discharge, the distribution shifted with an increase in the proportion of low burden (69.3%) and no burden (8.2%) patients, whereas the percentage of high burden patients decreased to 22.6%.

Although the median anticholinergic burden score was 2 at admission and remained 2 at discharge, a statistically significant reduction was observed (*p* < 0.001, Wilcoxon test). This reduction is reflected in the mean anticholinergic burden score, which decreased from 2.34 (±1.36) at admission to 1.82 (±1.26) at discharge. Overall, 160 patients (50.2%) experienced a reduction in their ACB score, whereas 93 (29.2%) had no change, and 66 (20.7%) had an increase in their score during hospitalisation. A detailed breakdown of the score changes is provided in Table 2.

**TABLE 2:** Change in anticholinergic burden score per patient between admission and discharge (*n* = 319).

Score change (discharge–admission)	*n* (%)
Reduction (total)	160 (50.2%)
−5 points	1 (0.3%)
−4 points	14 (4.4%)
−3 points	8 (2.5%)
−2 points	49 (15.4%)
−1 point	88 (27.6%)
No change	93 (29.2%)
0 points	93 (29.2%)
Increase (total)	66 (20.7%)
+1 point	38 (11.9%)
+2 points	22 (6.9%)
+3 points	4 (1.3%)
+4 points	2 (0.6%)

Metoclopramide was the most frequently prescribed anticholinergic agent at admission and was present in 69% of prescriptions. Only two medications with high anticholinergic activity (Level 3) were identified upon admission: amitriptyline (0.3%) and dimenhydrinate (1.9%). The overall reduction in anticholinergic burden score was primarily driven by the discontinuation of Level 1 medications, most notably metoclopramide (220 prescriptions at admission vs. 2 at discharge). Other significant discontinuations included morphine (46 vs. 0), isosorbide dinitrate (63 vs. 20) and clonazepam (35 vs. 3). Furthermore, dimenhydrinate, a high‐burden (Level 3) medication, was discontinued for all six patients who were prescribed it upon admission.

Conversely, new prescriptions or increases in use were also observed, which helps explain the increase in anticholinergic burden score seen in 66 (20.7%) patients. The most significant increases involved Level 1 medications, including metoprolol (from 159 to 221 prescriptions), colchicine (from 4 to 106) and codeine/paracetamol (from 0 to 12). Increases were also noted for higher burden agents, such as amitriptyline (Level 3, from 1 to 3) and carbamazepine (Level 2, from 1 to 2).

At discharge, there was a continued predominance of Level 1 anticholinergic medications. Metoprolol was the most frequently prescribed drug at this stage, accounting for 69.3% of prescriptions (Table 3). Anticholinergic burden was thus predominantly associated with medications classified as Level 1 on the Anticholinergic Activity Scale.

**TABLE 3:** Top 10 drugs prescribed and anticholinergic activity level.

Admission			
ATC	Drug	*n* (%)	Anticholinergic activity
N06AA09	Amitriptyline	1 (0.3)	3
R06AA11	Dimenhydrinate	6 (1.9)	3
C01AA05	Digoxine	7 (2.2)	2
N05AH04	Quetiapine	6 (1.9)	2
N03AF01	Carbamazepine	1 (0.3)	2
A03FA01	Metoclopramide	220 (69.0)	1
C07AB02	Metoprolol	159 (49.8)	1
C03CA01	Furosemide	125 (39.2)	1
C01DA08	Isosorbide	63 (19.8)	1
N02AA01	Morphine	46 (14.4)	1

Discharge			
ATC	Drug	*n* (%)	Anticholinergic activity
N06AA09	Amitriptyline	3 (0.9)	3
N05AH04	Quetiapine	4 (1.3)	2
N03AF01	Carbamazepine	2 (0.6)	2
C07AB02	Metoprolol	221 (69.3)	1
M04AC01	Colchicine	106 (33.2)	1
C03CA01	Furosemide	100 (31.4)	1
B01AA03	Warfarin	31 (9.7)	1
C01DA08	Isosorbide	17 (5.3)	1
C02DB02	Hydralazine	17 (5.3)	1
N02AJ06	Codeine and Acetaminophen	12 (3.8)	1

Univariate analysis at admission showed a significant association between polypharmacy (PR = 2.21; 95% CI = 1.61–3.04), the use of potentially inappropriate medications (PR = 5.00; 95% CI = 2.32–10.79) and a high anticholinergic burden, and the final multivariate analysis indicated polypharmacy (PR = 2.00; 95% CI = 1.47–2.72) and the use of potentially inappropriate medications (PR = 4.47; 95% CI = 2.10–9.53) remaining independently associated with a higher anticholinergic burden (Table 4).

**TABLE 4:** Patients' factors associated with anticholinergic burden at admission, *n* = 302^a^

Variable	Low/moderate burden *n* (%)	High burden *n* (%)	PR (95% CI)	*p*	Adjusted PR (95% CI)	*p*
Sex						
Male	111 (61.3)	70 (38.7)				
Female	64 (52.9)	57 (47.1)	1.22 (0.94, 1.58)	0.155	1.17 (0.92, 1.49)	0.205
Literacy						
Literate	138 (59.2)	95 (40.8)				
Illiterate	37 (53.6)	32 (46.4)	1.14 (0.85, 1.53)	0.409	—	—
Poor self‐rated health						
No	52 (59.8)	35 (40.2)				
Yes	123 (57.2)	92 (42.8)	1.06 (0.79, 1.43)	0.701	—	—
Poor self‐rated memory						
No	123 (56.4)	95 (43.6)				
Yes	52 (61.9)	32 (38.1)	0.87 (0.64, 1.19)	0.436	—	—
Medication use before admission						
No	19 (57.6)	14 (42.4)				
Yes	156 (58.0)	113 (42.0)	0.99 (0.65, 1.51)	1.000	—	—
ADL impairment						
No	157 (58.4)	112 (41.6)				
Yes	18 (54.5)	15 (45.5)	1.09 (0.73, 1.63)	0.711	—	—
Age group						
60–69 years	97 (57.1)	75 (42.9)				
≥ 70 years	78 (59.1)	54 (40.9)	0.95 (0.73, 1.25)	0.726	—	—
Polypharmacy type						
Polypharmacy	103 (74.6)	35 (25.4)				
Excessive polypharmacy	72 (43.9)	92 (56.1)	2.21 (1.61, 3.04)	< 0.001	2.00 (1.47, 2.72)	< 0.001
Dementia diagnosis						
No	150 (58.4)	107 (41.6)				
Yes	25 (55.6)	20 (44.4)	1.07 (0.75, 1.53)	0.745	—	—
Inappropriate prescribing						
No	54 (90.0)	6 (10)				
Yes	121 (50.0)	121 (50.0)	5.00 (2.32, 10.79)	< 0.001	4.47 (2.10, 9.53)	< 0.001

^a^ Patients without anticholinergic burden and those without polypharmacy were excluded from the analysis.

Univariate analysis at discharge also indicated that excessive polypharmacy (PR = 2.18; 95% CI = 1.37–3.47) and the prescription of potentially inappropriate medications (PR = 2.70; 95% CI = 1.78–4.07) were significantly associated with a higher anticholinergic burden, with age (≥ 70 years) inversely associated with outcome (PR = 0.70; 95% CI = 0.45–1.08). However, multivariate analysis indicated that only the prescription of potentially inappropriate medications remained significantly associated (PR = 2.39; 95% CI = 1.54–3.71) at this point (Table 5).

**TABLE 5:** Patients' factors associated with anticholinergic burden at discharge, *n* = 258^a^

Variable	Low/moderate burden *n* (%)	High burden *n* (%)	PR (95% CI)	*p*	Adjusted PR (95% CI)	*p*
Sex						
Male	119 (74.4)	41 (25.6)				
Female	71 (72.4)	27 (27.6)	1.08 (0.71, 1.63)	0.772	—	—
Literacy						
Literate	147 (74.6)	50 (25.4)				
Illiterate	43 (70.5)	18 (29.5)	1.16 (0.74, 1.83)	0.511	—	—
Poor self‐rated health						
No	59 (80.8)	14 (19.2)				
Yes	131 (70.8)	54 (29.2)	1.52 (0.90, 2.56)	0.117	1.43 (0.88, 2.32)	0.147
Poor self‐rated memory						
No	136 (73.5)	49 (26.5)				
Yes	54 (74.0)	19 (26.0)	0.98 (0.62, 1.55)	1.000	—	—
Medication use before admission						
No	19 (73.1)	7 (26.9)				
Yes	171 (73.7)	61 (26.3)	0.98 (0.50, 1.91)	1.000	—	—
ADL impairment						
No	174 (75.0)	58 (25.0)				
Yes	16 (61.5)	10 (38.5)	1.54 (0.90, 2.63)	0.160	1.67 (0.97, 2.86)	0.062
Age group						
60–69 years	101 (69.7)	44 (30.3)				
≥ 70 years	89 (78.8)	24 (21.2)	0.70 (0.45, 1.08)	0.118	0.74 (0.48, 1.13)	0.164
Polypharmacy type at discharge						
Polypharmacy	180 (75.9)	57 (24.1)				
Excessive polypharmacy	10 (47.6)	11 (52.4)	2.18 (1.37, 3.47)	0.008	1.57 (0.97, 2.56)	0.067
Dementia diagnosis						
No	164 (73.5)	59 (26.5)				
Yes	26 (74.3)	9 (25.7)	0.97 (0.53, 1.78)	1.000	—	—
Inappropriate prescribing						
No	183 (76.6)	56 (23.4)				
Yes	7 (36.8)	12 (63.2)	2.70 (1.78, 4.07)	< 0.001	2.39 (1.54, 3.71)	< 0.001

^a^ Patients without anticholinergic burden and those without polypharmacy were excluded from the analyses.

## 4 Discussion

This study evaluated changes in anticholinergic burden among hospitalised older adults with CVDs before and after a clinical pharmacist‐led medication review. The findings suggest a positive impact for pharmacist‐led medication review during hospitalisation, as evidenced by the reduced high anticholinergic burden from 40.4% at admission to 22.6% at discharge. Despite this reduction, most patients remain exposed to medications with some degree of anticholinergic activity. Hospitalisation should also be viewed as an opportunity to adjust treatment and medication dosage; however, inappropriate management in this context may increase mortality [26]. Deprescribing, which is legally equivalent to prescribing, requires the same standards of informed consent and patient monitoring [27].

Our findings are consistent with international evidence showing that pharmacist‐led medication reviews substantially reduce anticholinergic burden among hospitalised older adults. In the systematic review [12], the magnitude of reduction varied across individual studies, reflecting differences in metrics and intervention designs, but all demonstrated meaningful decreases in exposure to strong anticholinergic drugs in Europe, Australia and South Korea. Notably, our study provides the first evidence from South America, extending the global relevance of these interventions and reinforcing their effectiveness across diverse healthcare contexts.

Metoclopramide was the most frequently prescribed medication with anticholinergic properties at admission, which is consistent with the findings of a previous study involving hospitalised older adults with cancer [28]. One possible explanation for this result is the prevalence of nausea and vomiting in both cardiovascular and oncologic patient populations. Metoprolol was the most frequently prescribed anticholinergic agent at discharge. This may be explained by the fact that beta‐blockers are indicated for the management of symptomatic coronary artery disease, stable heart failure with reduced ejection fraction and chronic atrial fibrillation with an uncontrolled heart rate [29].

In the present study, nearly all prescriptions with high anticholinergic burden at admission and a notable proportion at discharge included at least one potentially inappropriate medication. Potentially inappropriate prescription was strongly associated with a high anticholinergic burden at both time points. This result was expected considering that medications with a high anticholinergic burden are considered inappropriate for older adults. Moreover, over one‐third of the drugs included in the Brazilian Anticholinergic Activity Scale used in this study are also listed in the 2015 American Geriatrics Society Beers Criteria (which served as the basis for the Brazilian scale) [6].

A high prevalence of polypharmacy was observed at both admission and discharge. Polypharmacy, a common geriatric syndrome, is highly prevalent in older adults and is strongly associated with adverse outcomes such as falls, mood disturbances and cognitive and functional decline [30, 31]. With the growing availability of cardiovascular medications, polypharmacy may be clinically justified when all prescribed drugs align with the current best evidence guidelines [32]. Thus, the notion that polypharmacy is inherently harmful or indicative of poor clinical practice should be reconsidered, particularly in the context of evidence‐based therapeutic decisions that are tailored to the needs of individual patients [33]. However, inappropriate polypharmacy, the use of medications that are potentially excessive, unnecessary, ineffective or harmful, poses a significant risk to older individuals and is thus of particular concern [32].

Excessive polypharmacy was associated with a high anticholinergic burden upon admission. Although polypharmacy and anticholinergic burden are distinct concepts, they are closely interrelated [3]. Consistent with earlier findings [3, 4], our results confirmed that higher anticholinergic scores are often linked to increased medication use, underscoring the need for cautious evidence‐based prescribing. Furthermore, although medications with high anticholinergic scores were key contributors to the overall burden, the predominance of medications with Level 1 anticholinergic activity in our study shows that the cumulative effect of multiple drugs with lower anticholinergic scores also played a substantial role.

The analysis of factors associated with a high anticholinergic burden revealed a notable shift from admission to discharge, likely reflecting the impact of clinical pharmacist interventions. At admission, both excessive polypharmacy and the presence of potentially inappropriate medication were strongly associated with a high anticholinergic burden. However, only potentially inappropriate medication remained a significant factor at discharge, and the strength of the association decreased. This shift suggests that pharmacist‐led interventions were effective in addressing polypharmacy and the most evident instances of inappropriate prescribing. Consequently, the group of patients who remained with a high anticholinergic burden upon discharge may represent more complex or severe clinical cases, in which the benefits of certain anticholinergic medications were deemed to outweigh the risks. This could also explain the reduced strength of the association for potentially inappropriate medication at discharge; as the most clearly inappropriate medications were deprescribed, the remaining potentially inappropriate medication contributing to the anticholinergic burden may have had a more nuanced or justifiable clinical indication. Regarding other factors such as sex, no statistically significant association was found, which might be attributable to the non‐probabilistic sampling design of the study.

The anticholinergic medications most prescribed at discharge are frequently prescribed for the treatment of CVDs. However, several barriers remain in deprescribing cardiovascular drugs, such as adverse withdrawal effects, limited data on the optimal treatment duration, time to benefit or harm and the effectiveness of cardiovascular medications in older adults with multimorbidity [33]. In contrast, deprescribing has proven to be feasible and generally safe, with minimal impact on adverse outcomes such as hospitalisations, falls and cardiovascular events in older adults with cardiometabolic conditions [34]. In terms of the continuum of safe prescription, cardiovascular teams should recognise deprescribing as a valuable strategy to improve the care and quality of life in older patients [34]. Clinical trials have shown that pharmacist‐led medication reviews conducted in collaboration with physicians promote deprescribing in various practice settings [35]. Our findings highlight the importance of implementing structured medication reviews during hospitalisation to promote safer prescribing practices.

## 5 Strengths and Limitations

This study has several important strengths, including its focus on a specific high‐risk population, a validated anticholinergic burden scale that is adapted to the Brazilian context and real‐world clinical data collected at a reference hospital. A key methodological strength of this study was the use of the Brazilian Anticholinergic Activity Scale. Although the multiplicity of anticholinergic scales presents a general challenge for comparability across studies, our choice of scale represents a robust solution in the context of the study. This scale was developed by synthesising several international instruments, better tailoring it to the pharmacological reality of medications prescribed to the Brazilian population. Furthermore, its reported good agreement with major international scales such as the Anticholinergic Drug Scale and Anticholinergic Cognitive Burden Scale enhances the validity and comparability of our estimates [36].

However, several limitations that are inherent to secondary data analysis and the before‐and‐after design of the original study should be considered when interpreting the results. First, although the use of the Brazilian Anticholinergic Activity Scale was a strategic choice, the inherent heterogeneity among existing anticholinergic scales warrants caution when comparing our results with those of other studies. Second, using data that were initially collected for a different primary research objective may restrict the scope of the available variables and limit the ability to control for potential confounding factors. Moreover, the secondary nature of the analysis precludes control over the data collection process, preventing verification of the quality, consistency and completeness of the data. Additionally, no control group was included in the original study, meaning that the effects of the pharmacist intervention from other time‐related or contextual factors, such as changes in clinical protocols or prescribing habits, cannot be isolated. Furthermore, symptoms potentially related to anticholinergic burden, cognitive impairment, delirium and ADL impairment were assessed only at the time of admission.

## 6 Conclusion

In conclusion, this study suggests that hospital‐based medication reviews and deprescribing support from clinical pharmacists can help reduce the anticholinergic burden in older adults with CVDs, reinforcing the importance of their integration into inpatient care to improve prescribing safety. Future research should assess the long‐term clinical outcomes associated with reductions in anticholinergic burden, such as cognitive function improvements, reduced adverse drug events and hospital readmissions. Additionally, comparative studies using different anticholinergic burden scales could clarify the implications of scale selection and improve standardisation in clinical research.

## Author Contributions

Luiz Carlos Santana Passos contributed to the study conception and design. Romana Santos Gama and Marcio Galvão Oliveira contributed to the study conception and design, material preparation, data collection and analysis and were the primary contributors in drafting the manuscript. Welma Wildes Amorim and Renato Morais Souza drafted the manuscript and critically reviewed the content. All the authors provided feedback on the previous manuscript and approved the final version.

## Funding

This study was partially financed by the Coordenação de Aperfeiçoamento de Pessoal de Nível Superior, Brazil (CAPES), Finance Code 001.

## Conflicts of Interest

The authors declare no conflicts of interest.

## Data Availability

The data that support the findings of this study are available on request from the corresponding author. The data are not publicly available due to privacy or ethical restrictions.
